# The role of neutrophil extracellular traps in Crohn's disease

**DOI:** 10.1016/j.heliyon.2024.e40577

**Published:** 2024-11-20

**Authors:** Ying Liu, Heng Deng, Jinfeng Yao, Chunrong He, Jun Zhang

**Affiliations:** a College of Chinese Medicine, Anhui University of Chinese Medicine, Hefei, Anhui, China; b Department of Anorectal Surgery, The Second Affiliated Hospital of Anhui University of Chinese Medicine, Hefei, Anhui, China; c Department of Internal Medicine, Anhui Hospital Affiliated Shanghai Shuguang Hospital, Hefei, Anhui, China; d Hefei Haiheng Health Service Center, Hefei, Anhui, China

**Keywords:** Neutrophil extracellular traps, Crohn's disease, Inflammation, Pathogenesis

## Abstract

Crohn's disease (CD) is an idiopathic and chronic inflammation of the gastrointestinal (GI) tract. The underlying pathogenesis of CD is multifaceted, with complex interactions between genetic predisposition, environmental triggers, and abnormalities within the immune system. Neutrophil extracellular traps (NETs) have gained significant attention as a novel component in the pathogenesis of CD. NETs are intricate structures fashioned from DNA, histones, and granule proteins, and are actively released by neutrophils to entangle and eliminate pathogenic microbes. This review article delves into the intricate role of NETs in the pathogenesis of CD. We examine how NETs may serve as a pivotal mechanism for the recruitment of immune cells to the site of inflammation. NETs are known to influence the function of epithelial cells, which line the GI tract, potentially contributing to the structural integrity and barrier dysfunction observed in CD. NETs stimulate inflammation, a hallmark of the disease, by releasing pro-inflammatory molecules and activating immune cells. We also investigate the promising therapeutic potential of targeting NETs in CD. By intercepting the formation or function of NETs, it may be possible to mitigate the chronic inflammation, reduce tissue damage, and alleviate the symptoms associated with CD. Strategies to inhibit NET formation, such as the use of DNase I and approaches to disrupt NET-mediated signaling pathways, are discussed in CD therapeutics. Understanding the detailed mechanisms of NETs is crucial for the development of targeted treatments that could potentially revolutionize the management of CD.

## 1 Introduction

Crohn's disease (CD) is a prototypical inflammatory bowel disease that exhibits a heterogeneous clinical presentation, ranging from quiescent disease with few symptoms to fulminant disease requiring urgent surgical intervention [1]. The hallmark of CD is chronic inflammation that persists over time [2], affecting any part of the gastrointestinal (GI) tract from the mouth to the anus [3]. This chronic inflammation is characterized by cycles of exacerbation and remission [4], leading to significant morbidity and mortality in affected patients [5]. The pathogenesis of CD is a complex interplay of genetic susceptibility [6], environmental triggers [7], and aberrant immune responses [8]. Genome-wide association studies have identified numerous genetic loci associated with CD risk, suggesting a strong genetic component to the disease [9]. In CD, environmental factors, such as infections [10], diet [11], and gut microbiota [12], have also been implicated in the initiation and perpetuation of inflammation that was associated with neutrophil extracellular traps (NETs).

NETs are a recently discovered component of the innate immune response [13], which plays a critical role in the initial host defense against invading pathogens [14]. NETs are intricate structures composed of DNA, histones, and various granule proteins, primarily released by neutrophils in response to microbial recognition or inflammation-induced cell death [15,16]. Initially thought to be a tool for microbial clearance, NETs are now recognized to have diverse functions in both health and disease, including the regulation of inflammation [17], cell death [18], and tissue repair [19]. In the context of CD, NETs have emerged as pivotal players in the pathogenesis of the disease [20]. They contribute to the chronic inflammation and tissue damage observed in CD through several mechanisms [21].

This review aims to comprehensively discuss the mechanism of action of NETs in Crohn's disease, highlighting their role in the initiation and maintenance of chronic inflammation, tissue damage, and the complex interplay between the host immune system and gut microbiota. Understanding the precise mechanisms by which NETs contribute to CD pathogenesis is critical for the development of novel diagnostic and therapeutic strategies that target the underlying causes of the disease, rather than just the symptoms.

## 2 The formation of NETs

**Fig. 1:**
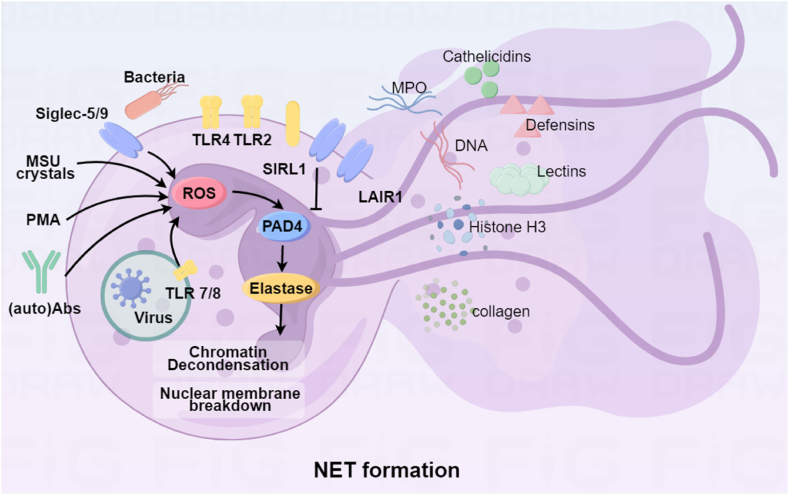
The NET formation and composition: Neutrophils turn on NETosis after receiving the corresponding stimulus. LAIR1: Interleukin-associated immunoglobulin-like receptor-1; TLR7/8: Toll-like receptors; SIRL-1: Leukocyte signal suppressor Receptor-1; Siglec-5/9: Sialic acid-binding immunoglobulin-like lectins-5/9; MSU crystals: Sodium urate crystals; PMA: 2-Acetoxy-1-methoxypropane. PAD4: peptidyl arginine deiminase 4.

NETs represent a novel aspect of the innate immune response, characterized by their intricate web-like structures that serve as a platform for host defense and inflammation [22]. These structures are primarily composed of DNA, histones, and a variety of granule proteins. The DNA in NETs is not continuous but rather fragmented into shorter strands, which are then complexed with histones, forming a scaffold-like structure [23]. This scaffold provides the physical foundation for the recruitment and assembly of granule proteins, creating a matrix that can entangle pathogens and facilitate their clearance by the immune system [24]. The granule proteins that are incorporated into NETs include a diverse array of enzymes and toxic molecules that are typically stored within neutrophil granules [25]. These proteins include elastase [26], myeloperoxidase (MPO) [27], neutrophil gelatinase B (NGAL), peptidyl arginine deiminase 4 (PAD4), and proteinase 3 (PR3), among others [28] (Fig. 1).

## 3 The composition of NETs

Elastase is an enzyme that can degrade elastin, a key component of the extracellular matrix, which is important for the structural integrity of tissues [29]. PAD4 converts arginine from histones to citrulline [30], which is involved in the process of chromatin decoagulation and extension, and causes DNA to form a network of chromatin [31]. This is the structural basis of NETS. MPO, in conjunction with hydrogen peroxide, can produce reactive oxygen species (ROS) that have antimicrobial properties and can also contribute to tissue damage [32]. PR3 is another enzyme that can contribute to tissue damage by breaking down structural proteins [33]. In addition to these enzymes, NETs also contain other proteins, such as defensins, cathelicidins, and lectins, which have antimicrobial properties and can directly kill pathogens [34]. The presence of these proteins on the surface of NETs allows them to interact with pathogens and facilitate their immobilization and subsequent destruction. NETs are not static structures; they are highly dynamic and can evolve over time. The ability of NETs to recruit and activate various components of the immune system underscores their potential role in the pathogenesis of various diseases [35], including inflammatory disorders like Crohn's disease.

## 4 NETs in CD pathogenesis

### 4.1 Chronic inflammation

In CD, NETs contribute to the chronic inflammation observed in the gastrointestinal tract [36]. NETs can entangle bacteria and fungi, promoting their presentation to the immune system and the activation of innate and adaptive immune responses [37]. This leads to the recruitment of immune cells, such as macrophages and T cells [38], and the release of pro-inflammatory cytokines and chemokines, such as tumor necrosis factor-alpha (TNF-α) and interleukin-1β (IL-1β), exacerbating the inflammatory response [39]. This mechanism is also present in the occurrence of complex perianal fistulizing Crohn's Disease [40]. NETs can also activate the complement system [41], which can lead to the recruitment of additional immune cells and the release of anaphylatoxins, such as C5a, which can exacerbate inflammation [42].

### 4.2 Tissue damage

NETs can directly contribute to tissue damage in CD. The granule proteins present in NETs, such as elastase and myeloperoxidase, can degrade extracellular matrix components, leading to epithelial barrier dysfunction and increased intestinal permeability [43]. This allows the entry of harmful substances and pathogens into the epithelial layer, further promoting inflammation and tissue damage.

### 4.3 Microbial evasion and Adherence

Some gut microbes in CD can evade or adhere to NETs, contributing to the development of persistent infections. This can lead to the development of antibiotic resistance and the persistence of pathogens that promote inflammation and tissue damage [44].

### 4.4 Autoimmunity

NETs can also contribute to the development of autoimmunity in CD [45]. NETs can promote the activation and proliferation of autoreactive T cells and B cells, leading to the production of autoantibodies and the development of chronic inflammation[36,46].

### 4.5 Modulation on epithelial cell function

NETs can also modulate epithelial cell function in CD. Neutrophil-derived extracellular vesicles carrying PAD4 were secreted into intestinal epithelial cells, in which, PAD4 reduced the stability of mitochondrial creatine kinase 1 protein via the autophagy pathway [47]. These changes can contribute to the fibrosis and stricture formation observed in CD.

## 5 Therapeutic implications of targeting NETs in CD

Given the role of NETs in the pathogenesis of CD, targeting NETs has become a promising therapeutic strategy. inhibitors of NET formation, such as DNase I and neutralizing antibodies against NET components, have been shown to reduce inflammation and improve symptoms in animal models of CD [21]. Additionally, inhibitors of NET-associated signaling pathways, such as the inhibition of complement receptor type 3 (CR3) and platelet-derived growth factor (PDGFRα), have been shown to reduce inflammation and fibrosis in animal models of CD [48]. These findings suggest that targeting NETs may represent a novel and effective therapeutic approach for the treatment of CD.

## 6 Discussion

The pathogenesis of CD is a complex interplay of genetic, environmental, and immune system factors, which culminates in chronic inflammation, tissue damage, microbial evasion, and autoimmunity [49]. NETs have emerged as key players in these processes, contributing to the ongoing inflammation and subsequent tissue destruction observed in CD [50]. NETs, consisting of DNA, histones, and granule proteins, are released by neutrophils to immobilize and kill microbes [51]. However, in CD, NETs appear to have a more nuanced role, promoting chronic inflammation by recruiting immune cells, such as macrophages and T cells, to the site of infection or injury [52]. This recruitment is mediated through the interaction of NET components with cell surface receptors, such CR3 and PDGFRα [53]. Furthermore, NETs can promote microbial evasion by trapping bacteria within their structure, preventing their clearance by the immune system [54]. This microbial persistence can lead to a breakdown of the host-microbial balance and the development of a dysregulated immune response, contributing to the autoimmunity observed in CD [55]. In ulcerative colitis, also an inflammatory bowel disease, the causative mechanism of NETs is roughly similar to that of CD, but NETs have shown beneficial effects in serious diseases such as necrotizing enterocolitis and hemorrhagic colitis [56].

Given the central role of NETs in the pathogenesis of CD, targeting NETs represents a promising therapeutic strategy. In the initial inflammatory phase of CD, inhibition of NET formation, through the use of drugs such as DNase I [57], or the neutralization of NET components with antibodies, has been shown to reduce inflammation and tissue damage in animal models of CD [21]. During the intermediate stages, while NETs intensify inflammation, fibrotic development is not yet fully consolidated. At this juncture, alongside therapies that directly dismantle NETs, the incorporation of targeting NET-associated signaling pathways, such as CR3 and PDGFRα, holds potential for modulating the immune response and preventing fibrotic changes in the gut [58]. In the advanced stages, where fibrosis has become established, it is pertinent to continue with NET-degrading treatments like DNase I, and also to consider the introduction of anti-fibrotic agents, such as TGF-β inhibitors, to ameliorate the extent of fibrosis [59].

However, the precise mechanisms by which NETs contribute to the pathogenesis of CD are still not fully understood. The interactions between NETs, other immune cells, and the gut microbiota are complex and require further investigation [60]. Moreover, the clinical translation of NET-targeting therapies requires careful consideration of potential side effects and the balance between reducing inflammation and maintaining a protective immune response against pathogens [61].

In conclusion, NETs are potential therapeutic targets could be strengthened and more emphasis could be placed on specific future research.

## CRediT authorship contribution statement

**Ying Liu:** Writing – original draft. **Heng Deng:** Funding acquisition, Data curation, Conceptualization. **Jinfeng Yao:** Formal analysis, Data curation. **Chunrong He:** Visualization, Supervision, Methodology. **Jun Zhang:** Resources, Project administration.

## Ethics declarations

In this review, references to human studies have been made to ensure that the studies have obtained the appropriate ethical approval and informed consent of the participants. When quoting and analyzing literature, we respect the privacy of all research subjects and do not disclose any information that may lead to identification; Ensure that the cited research methods and results are rigorously validated; Strictly abide by the principles of legality and transparency of data use.

## Data availability statement

Data included in article/supp. material/referenced in article.

## Funding

This research was funded by Scientific Research Project of Colleges and Universities of Anhui Province (No. 2022AH050442, 2023AH050848, and 2024AH051038); and Xinglin talent training program (No. 0500-48-30).

## Declaration of Competing Interest

The authors declare the following financial interests/personal relationships which may be considered as potential competing interests:Heng Deng reports financial support was provided by Scientific Research Project of Colleges and Universities of Anhui Province. If there are other authors, they declare that they have no known competing financial interests or personal relationships that could have appeared to influence the work reported in this paper.

## Abbreviations

NETs: analysis of variance
CD: Crohn's disease
GI: gastrointestinal
MPO: myeloperoxidase
NGAL: neutrophil gelatinase B
PAD4: peptidyl arginine deiminase 4
PR3: proteinase 3
ROS: reactive oxygen species
TNF-α: tumor necrosis factor-alpha
IL: interleukin
CR3: Complement receptor type 3
PDGFRα: platelet-derived growth factor
